# Supplementary material for: Optimization of Temporal Coding of Tactile Information in Rat Thalamus by Locus Coeruleus Activation
Source: Biology (Basel). 2024 Jan 28;13(2):79. doi: 10.3390/biology13020079 (PMC10886390; doi:10.3390/biology13020079)
Supplement: Supplementary file 1 [file biology-13-00079-s001.zip › biology-2809815-SI.pdf]

Supplementary Materials:

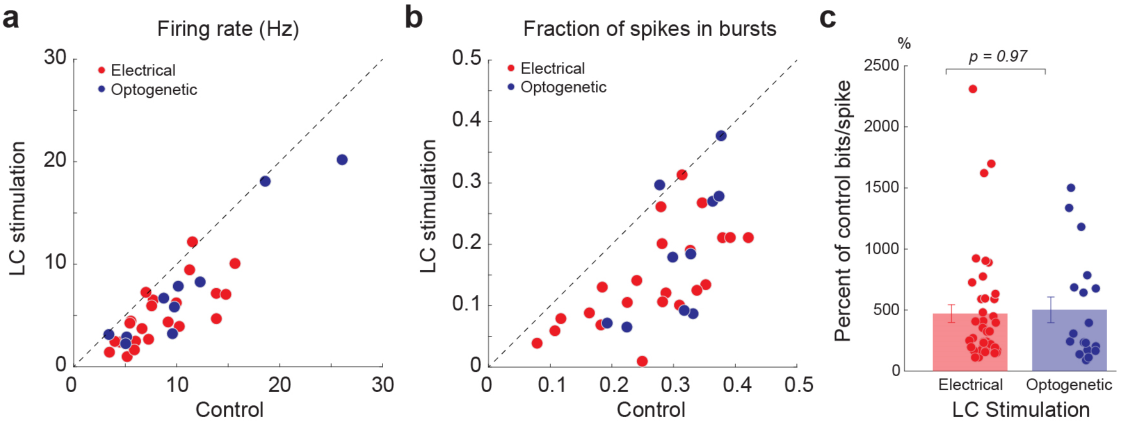

Supplemental Figure S1. No significant differences in effects of electrical and optogenetic LC stimulation on thalamic responses. (a) Firing rate with and without LC activation. (b) Fraction of spikes in bursts with and without LC activation. (c) Improvement in information transmission with electrical and optogenetic LC stimulation.

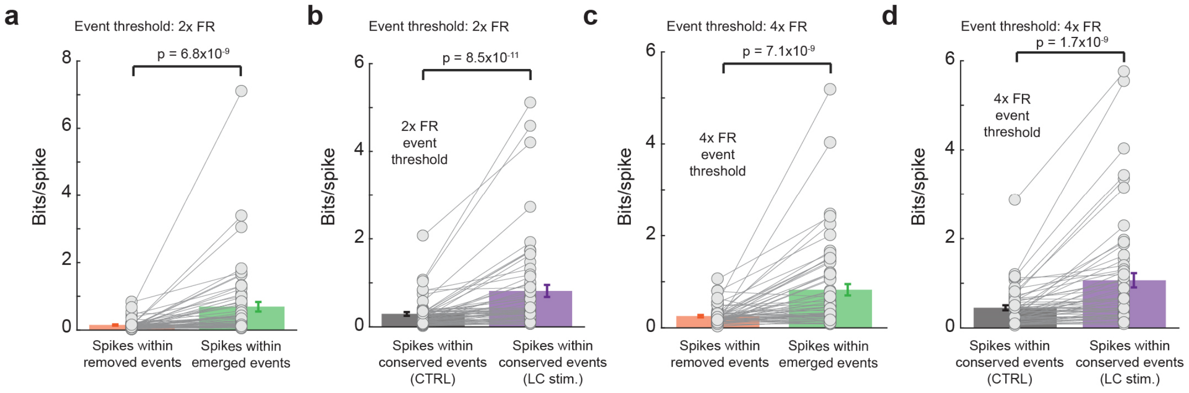

Supplemental Figure S2. Different event thresholds resulted in quantitatively similar results. (a) Information transmission for spikes within removed events vs. spikes within emerged events using the event threshold of 2x firing rate. (b) Information transmission for spikes within conserved events without LC stimulation vs. spikes within conserved events during LC stimulation using the event threshold of 2x firing rate. (c) Information transmission for spikes within removed events vs. spikes within emerged events using the event threshold of 4x firing rate. (d) Information transmission for spikes within conserved events without LC stimulation vs. spikes within conserved events during LC stimulation using the event threshold of 4x firing rate.
